# Identifying sarcoidosis trends using web search and real-world data in Sweden: a retrospective longitudinal study

**DOI:** 10.1038/s41598-024-69223-8

**Published:** 2024-08-20

**Authors:** Stefanie Ziehfreund, Linda Tizek, Elizabeth V. Arkema, Alexander Zink

**Affiliations:** 1 Department of Dermatology and Allergy, Technical University of Munich, TUM School of Medicine and Health, Munich, Germany; 2 Clinical Epidemiology Division, Department of Medicine Solna, Karolinska Institutet, Stockholm, Sweden; 3 Division of Dermatology and Venereology, Department of Medicine Solna, Karolinska Institute, Stockholm, Sweden

**Keywords:** Incidence, Infodemiology, Sarcoidosis, Real-world data, Web search, Public health, Respiratory signs and symptoms, Skin manifestations

## Abstract

Web search data are associated with disease incidence, population interest, and seasonal variations. This study aimed to investigate seasonal and geographical variations of web search data for sarcoidosis and to explore its association with external factors and sarcoidosis incidence in Sweden. Therefore, sarcoidosis-related data from Google Ads Keyword Planer (2017–2020) were generated for Sweden according to its 21 counties. The relationship between search volume and season, region, population demographics, environmental factors, and the sarcoidosis incidence listed in the National Patient Register was assessed. Analyses revealed seasonal variations for Sweden with an overall peak in the spring and autumn. Geographical differences were observed, with a higher search volume for north-western counties and the lowest search volume for Stockholm County. At the country level, the search volume was positively associated with the sarcoidosis incidence. Higher male proportion and older mean age were associated with a higher search volume, while a higher proportion of foreign-born residents, humidity, and mean temperature were associated with a lower search volume. Our analyses detected correlations between web search data, sarcoidosis incidence, and external factors. Analyses of sarcoidosis web search data therefore appear to be a valuable approach to disease surveillance to address medical needs and public interest.

## Introduction

Sarcoidosis is a chronic, inflammatory disease characterized by non-caseating granulomas. Sarcoidosis is very heterogeneous in terms of onset, and it can manifest in almost any organ of the body including the skin, heart, joints, lymph nodes, and gastrointestinal tract, although the lungs are affected in more than 90% of affected individuals^1,2^. The aetiology and pathogenesis of sarcoidosis are largely unknown, and the prognosis depends on the type of disease, therapy response, and the affected individual’s general conditions^2,3^. The burden of sarcoidosis for individuals and society is substantial^2,4^. In addition to its economic and psychological impact, sarcoidosis is also characterized by its non-specific symptoms that complicate the diagnostic process^2,4^.

Incidence and prevalence estimates vary substantially depending on ethnicity and geography, with the highest sarcoidosis prevalence reported for African Americans and in Nordic countries like Sweden^5–7^. Higher rates of sarcoidosis in certain regions may be due to a common contagion, genetic predisposition, and environmental exposure. Local weather (e.g., temperature, sunshine hours, and humidity) is believed to be a key environmental factor influencing the incidence of sarcoidosis in a specific geographic area. Moreover, population density, occupational activities, household, and lifestyle determinants are discussed as environmental factors associated with the development of sarcoidosis^8–10^. While some studies did not show any gender differences^11,12^, others reported that sarcoidosis was more common in women^13,14^. A nationwide register-based study in Sweden, however, reported a higher incidence and prevalence in men compared to women (16.0 vs. 13.5/100,000 per year and 179 vs. 141/100,000, respectively)^6^. The observed peak in age of onset in Europe and the United States is between 20 and 60 years, occurring earlier in men than in women^2,6,9,13,14^.

Nowadays, internet search engines are widely used to seek health information or to evaluate symptoms. Moreover, the internet may play an essential role in rare diseases like sarcoidosis in information seeking^15^. Previous studies have revealed that search analysis—one of the methods of infodemiology and infoveillence^16^—can be a powerful tool to reflect a population’s interest in specific topics and thus can be associated with conventional data^17,18^. Numerous studies have also shown that web search data are a valuable resource for studying noncommunicable diseases and may be able to predict diseases^19–21^. Recent analyses of web search queries for sarcoidosis found seasonal and geographical variations and indicated an overall unmet medical need^22,23^. However, no study to date has assessed factors associated with sarcoidosis-related search behaviour to monitor sarcoidosis and related medical needs. In the present study, we analysed web search data on sarcoidosis in Sweden from January 2017 to December 2020 to examine seasonal and geographic trends. Furthermore, we examined the relationship between search volume and environmental and population-related factors as well as the sarcoidosis incidence listed in the National Patient Register (NPR).

## Materials and methods

### Data collection

#### Internet search approach

For this retrospective longitudinal study, we used Google Ads Keyword Planner (Google Ads) to generate web search data of the Swedish term for sarcoidosis “sarkoidos” and related keywords for the 21 Swedish counties.

Google Ads is primarily used to optimize marketing campaigns. As it can reflect a population’s interest in certain topics, it is also increasingly used to answer scientific questions^18,22^. Google Ads provides the most relevant keywords with their average monthly search volume (SV) for different regions and timeframes. Data are available from the four years before the day of the query. We assessed the SV across the Swedish counties from January 1, 2017, to December 31, 2020. Data were limited solely to users in Sweden whose language preference was Swedish. We then normalized the results per 100,000 inhabitants to account for different population sizes between counties. Numbers of inhabitants in the Swedish counties are provided in Additional file 1: Table S1 (www.statistikdatabasen.scb.se).

#### Sarcoidosis incidence from administrative health data

Data on date and ICD code for sarcoidosis diagnoses in Sweden were obtained from the NPR. The NPR includes data on both inpatient hospitalizations since 1964 (nationwide since 1987; coverage of nearly 100%) and outpatient visits since 2001 (coverage of approximately 87%). Visits have been coded using the Swedish version of the ICD coding system (code for sarcoidosis: ICD-8 135, ICD-9 135, or ICD-10 D86). We identified individuals receiving their first-ever visit listing ICD code for sarcoidosis occurring from January 1, 2010, to December 31, 2013.

#### Population statistics and environmental factors

Yearly data on inhabitant demographics (mean age, proportion of male inhabitants, foreign-born residents per 100,000 inhabitants, and population density (sq. km)) were taken from Statistics Sweden (Statistiska centralbyrån (SCB); www.statistikdatabasen.scb.se) for the years 2017 to 2020. Weather data (monthly mean humidity (%), sunshine duration (hours), and temperature (°C)) for all counties were taken from the Swedish Meteorological and Hydrological Institute (SMHI; www.smhi.se/data) for the years 2017 to 2020.

Institutional review board approval and informed consent were not applicable for the internet data. Ethical approval for the Swedish register data on the incidence of sarcoidosis was obtained from the Swedish Ethical Review Authority DNR 2014-230-31, amendment 2020-00437.

#### Statistical analysis

Descriptive data were generated for the SV of sarcoidosis for Sweden as a whole and for its counties (absolute SV per 100,000 and mean per 100,000, standard deviation (SD)). Keywords identified by Google Ads were qualitatively analysed. Differences in SV per 100,000 inhabitants between Sweden’s counties and European seasons (meteorological seasons; winter: December–February, spring: March–May, summer: June–August, and autumn: September–November), were tested with Kruskal–Wallis tests and Friedman tests, respectively, as SV in counties and seasons was not normally distributed. Dunn-Bonferroni corrector was used for post hoc analysis. Spearman’s correlation coefficient was used to assess the relationship between the monthly SV and (1) sarcoidosis incidence and (2) the aforementioned external factors. The comparison between sarcoidosis SV 2017–2020 and incidence 2010–2013 was justifiable, as studies have demonstrated that the incidence of sarcoidosis in Sweden has not increased in recent years ^6^ and that there were no significant differences over time in the number of new diagnoses in the available NPR dataset since January 2007 (p = 0.34). All factors were included in a multivariable linear regression model to further assess the relationship with SV. To avoid multicollinearity, correlations of all variables were calculated before inclusion in the multivariable model. Standardized regression coefficient (beta) and 95% confidential intervals (95%-CIs) were estimated. P values < 0.05 were considered statistically significant. Analyses were performed using IBM SPSS Statistics for Windows, version 28.0 (IBM Corp). Geodata from the European Commission—Eurostat/GISCO were used to determine administrative boundaries using a geographic information system, QGIS, version 3.30.3 (QGIS Development Team).

### Ethics statement

Institutional review board approval and informed consent were not applicable for the internet data. Ethical approval for the Swedish register data on the incidence of sarcoidosis was obtained from the Swedish Ethical Review Authority DNR 2014-230-31, amendment 2020-00437.

## Results

**Figure 1:**
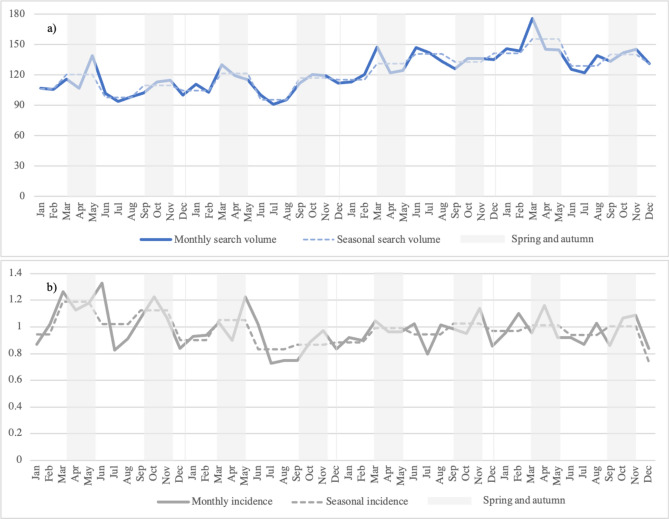
Monthly and seasonal sarcoidosis data per 100,000 inhabitants in Sweden. (**a**) Google Ads sarcoidosis-related search volume per 100,000 inhabitants from January 2017 to December 2020. (**b**) Monthly sarcoidosis incidence per 100,000 inhabitants from 2010 to 2013. season = mean for winter, spring, summer, and autumn per year.

The total SV across the 21 Sweden counties was 123,555.74 per 100,000 inhabitants (mean: 122.58, (SD 41.18)). The greatest monthly SV for Sweden was observed in March 2020 with 175.83 searches per 100,000 inhabitants (Fig. 1a), while the highest monthly sarcoidosis incidence was registered in June 2010 (1.22/100,000 inhabitants; (Fig. 1b)). The highest mean monthly SV per 100,000 inhabitants was observed for “sarcoidosis” (71.75 (SD 27.52)). In total, 30 keywords related to the Swedish word for sarcoidosis were identified. Most of them assigned to “manifestations and forms” of sarcoidosis (e.g., pulmonary sarcoidosis, chronic sarcoidosis; 20/30, 66.67%), followed by treatment-related queries (e.g., treatment sarcoidosis; 4/30, 13.33%; [Additional file 2: Table S2]).

### Seasonal variation

Overall, the SV per 100,000 inhabitants showed seasonal variations (*x*^*2*^ = 55.25, p < 0.001), with significantly more searches in spring (125.94 (SD 37.58)) and autumn (123.84 (SD 48.91)) than in the summer (116.69 (SD 39.88)) and winter months (118.83 (SD 36.18), 0.001 ≤ p ≤ 0.006). No differences were identified between summer and winter (p = 0.099) and autumn and spring (p = 1.000, (Fig. 1a)).

### County variation in monthly SV

The mean monthly SV per 100,000 inhabitants for Swedish counties ranged from 50.07 (SD 7.98) in Stockholm County to 182.97 (SD 56.77) in Västerbotten County and showed significant differences between counties (*x*^*2*^ = 504.09, p < 0.001). Post hoc analyses showed a significantly lower number of monthly search queries per 100,000 for Stockholm County (50.07 (SD 7.98)) compared to all other counties (0.001 < p ≤ 0.009) except for Västra Götaland (83.57 (SD 14.93), p = 1.000). Västerbotten (182.97 (SD 56.77)) had a significantly higher number of monthly search queries than all the other counties (0.001 ≤ p ≤ 0.009) except for Skåne (96.39 (SD 15.73)), Södermanland (98.49 (SD 20.43)), and Halland (103.59 (SD 18.73)).

### Population statistics

The SV per 100,000 inhabitants showed a moderate and weak positive correlation with the inhabitants’ mean age (r = 0.44, p < 0.001, (Table 1)) and the proportion of male inhabitants (r = 0.23, p < 0.001). In contrast, the number of foreign-born persons per 100,000 inhabitants and population density showed a good negative correlation with the overall SV (r = − 0.52, p < 0.001 and r = − 0.52, < 0.001, respectively).

**Table 1:** Association between the search volume and selected demographic and environmental factors for Sweden.

Covariates	Mean (SD)	Spearman correlation		Multiple linear regression*	
		r_s_	p	beta	95%-CI
Monthly incidence (n/100,000)	0.99 (0.65)	0.16	0.003	5.86	2.57; 9.16
Mean age (years)	42.08 (4.51)	0.44	< 0.001	0.19	− 0.28; 2.48
Male inhabitants (%)	50.46 (0.35)	0.23	< 0.001	8.87	2.13; 14.42
Population density (sq. km)	50.70 (75.03)	− 0.52	< 0.001	NA**	NA
Foreign-born residents/100,000	15,771.44 (4442.39)	− 0.52	< 0.001	− 0.01	− 0.006; − 0.004
Monthly humidity (%)	79.40 (10.57)	0.05	0.134	− 0.25	− 0.67; 0.18
Monthly temperature (°C)	7.76 (6.99)	− 0.12	< 0.001	− 0.60	− 1.06; − 0.13
Monthly sunshine duration (h)	158.49 (109.51)	− 0.03	0.417	0.01	− 0.05; 0.06

CI, confidential interval.

*Adjusted for the included variables.

**Excluded due to the high multicollinearity with the variable “foreign-born residents”.

### Environmental factors

No correlation was observed between SV and the monthly sunshine hours (r = − 0.03, p = 0.417) as well as between the SV and the monthly relative humidity (r = 0.05, p = 0.135; (Table 1)). Furthermore, the monthly mean temperature showed a weak negative correlation with the overall SV (r = − 0.12, p < 0.001). Correlations between the different environmental factors and SV on the county level were weak to moderate (− 0.34 ≤ r ≤ 0.28, 0.02 ≤ p ≤ 0.94; (Table 2)).

**Table 2:** Association between the search volume and the sarcoidosis incidence as well as selected environmental factors across Sweden’s counties.

Counties (monthly mean SV/100,000)	Monthly incidence (n/100,000)			Humidity (%)			Monthly temperature (°C)			Monthly sunshine duration (h)		
	Mean (SD)	r_s_	p	Mean (SD)	r_s_	p	Mean (SD)	r_s_	p	Mean (SD)	r_s_	p
Stockholm (50)	0.90 (0.23)	0.38	0.008	77.54 (11.12)	0.25	0.09	7.37 (6.88)	− 0.30	0.04	170.80 (117.31)	− 0.34	0.02
Uppsala (133)	1.10 (0.60)	− 0.21	0.15	76.61 (12.81)	0.24	0.10	7.99 (7.19)	− 0.34	0.02	170.80 (118.32)	− 0.29	0.05
Södermanland (99)	0.79 (0.41)	− 0.29	0.46	71.67 (11.18)	− 0.25	0.08	7.92 (6.99)	− 0.09	0.55	163.46 (108.72)	− 0.10	0.50
Östergötland (115)	1.12 (0.51)	0.17	0.25	79.35 (10.64)	0.07	0.62	8.07 (6.76)	− 0.10	0.49	163.45 (108.72)	− 0.10	0.43
Jöngköping (107)	1.03 (0.50)	0.20	0.17	82.03 (11.04)	0.28	0.06	7.53 (6.51)	− 0.16	0.28	142.02 (102.16)	− 0.26	0.08
Kronoberg (114)	0.80 (0.59)	0.32	0.02	81.30 (12.02)	0.13	0.39	8.17 (6.55)	− 0.14	0.34	142.02 (103.16)	− 0.12	0.41
Kalmar (118)	1.04 (0.59)	0.07	0.64	81.13 (8.68)	-0.17	0.26	8.92 (6.31)	0.22	0.13	142.03 (102.16)	0.11	0.48
Gotlands (155)	0.44 (0.82)	0.04	0.98	81.92 (8.51)	0.03	0.87	8.63 (6.22)	0.07	0.64	185.21 (127.54)	− 0.02	0.91
Blekinge (131)	0.76 (0.71)	0.19	0.18	82.19 (5.46)	0.07	0.64	9.83 (6.23)	− 0.04	0.81	182.06 (119.10)	− 0.10	0.49
Skåne (96)	1.10 (0.27)	− 0.14	0.34	78.79 (89.67)	− 0.03	0.87	10.11 (6.25)	− 0.08	0.57	152.72 (103.25)	− 0.05	0.76
Hallands (104)	0.70 (0.37)	− 0.14	0.33	81.62 (8.22)	− 0.03	0.85	9.79 (6.16)	− 0.23	0.11	142.02 (102.16)	− 0.15	0.31
Västra Götaland (84)	0.88 (0.23)	0.17	0.24	79.79 (11.13)	− 0.01	0.94	9.85 (6.40)	0.06	0.69	150.74 (100.46)	− 0.05	0.75
Värmlands (123)	0.92 (0.70)	− 0.16	0.28	79.97 (12.26)	0.07	0.62	7.35 (6.92)	− 0.10	0.49	162.97 (108.11)	− 0.09	0.57
Örebro (137)	1.00 (0.51)	− 0.21	0.14	76.93 (12.01)	− 0.07	0.64	8.41 (6.91)	− 0.13	0.42	162.97 (108.11)	− 0.13	0.40
Västmanlands (118)	1.05 (0.60)	− 0.06	0.67	79.30 (11.99)	0.02	0.88	7.94 (7.21)	− 0.03	0.86	162.97 (108.11)	− 0.03	0.85
Dalarns (125)	1.20 (0.63)	− 0.17	0.26	78.81 (10.57)	0.03	0.84	6.72 (7.62)	− 0.02	0.89	159.79 (107.65)	− 0.09	0.55
Gävleborg (121)	0.80 (0.58)	0.13	0.49	79.21 (9.70)	− 0.07	0.64	6.73 (6.93)	0.12	0.44	159.79 (107.65)	0.05	0.73
Västernorrland (152)	1.25 (0.70)	0.43	0.002	78.98 (10.22)	0.03	0.82	4.99 (7.80)	− 0.20	0.49	167.68 (118.30)	− 0.07	0.63
Jämtland (179)	1.52 (1.26)	0.06	0.70	80.33 (10.17)	− 0.25	0.09	4.03 (7.34)	0.03	0.82	141.57 (104.76)	0.02	0.94
Västerbotten (183)	1.32 (.66)	0.12	0.41	81.12 (9.20)	− 0.10	0.50	4.43 (7.82)	− 0.12	0.41	167.68 (118.30)	− 0.07	0.62
Norrbotten (124)	0.97 (.60)	− 0.01	0.93	78.77 (9.09)	− 0.08	0.59	8.17 (6.55)	− 0.08	0.57	135.57 (108.14)	0.10	0.48

SV, search volume; SD, standard deviation.

Spearman’s correlation coefficient (r_s_) was used to assess the correlation between search volume and selected environmental factors for Sweden’s counties.

### Association between monthly SV and incidence

**Figure 2:**
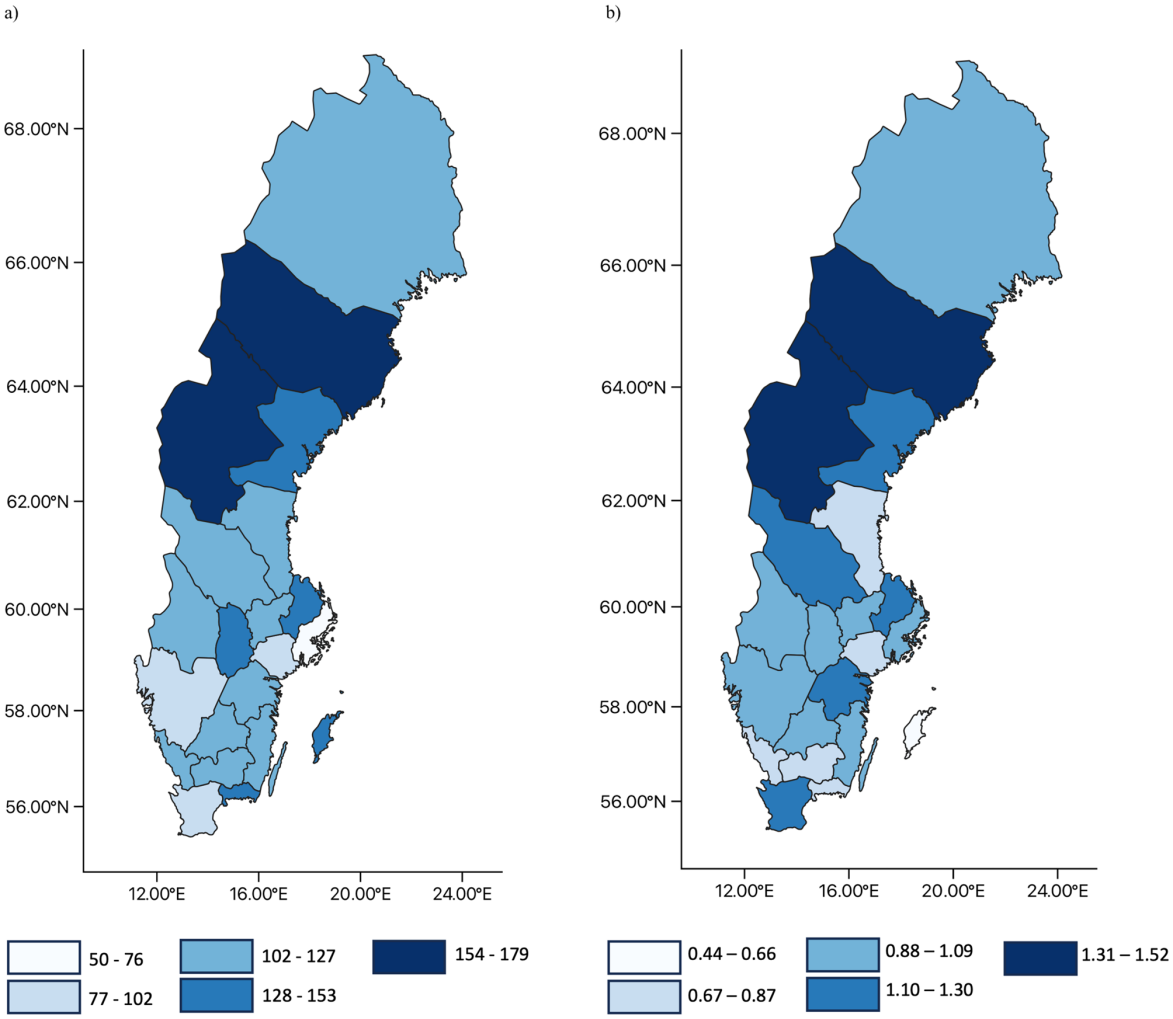
The monthly search volume and sarcoidosis incidence in 21 Swedish counties. (**a**) Swedish counties with the monthly search volume per 100,000 inhabitants for sarcoidosis from January 2017 to December 2020. (**b**) Swedish counties with the monthly sarcoidosis incidence per 100,000 inhabitants from January 2010 to December 2013.

Across Sweden, the monthly incidence showed a low positive correlation with the SV (r = 0.16, p = 0.003). Västerbotten, Jämtland, and Västernorrland, the counties with the highest sarcoidosis incidence per 100,000 inhabitants, exhibited elevated search frequencies compared to other counties (Fig. 2). On the county level, the SV showed a moderate correlation in Stockholm (r = 0.38, p = 0.008), Kronoberg (r = 0.32, p = 0.020), and Västernorrland (r = 0.43, p = 0.002; (Table 2)), while the other counties showed no to weak correlation (− 0.29 ≤ r ≤ 0.20, 0.14 ≤ p ≤ 0.98).

### Prediction of the sarcoidosis SV

Test for multicollinearity showed a correlation between a higher proportion of foreign-born residents and higher population density (r = 0.70, p < 0.001; [Additional file 3: Table S3]). Overall, the proportion of male inhabitants had the greatest effect on the SV per 100,000 inhabitants, with a higher proportion resulting in a higher SV (beta 8.87, 95%-CI [2.13; 14.42], p = 0.005, (Table 1)). A higher monthly sarcoidosis incidence, a higher mean age, and more sunshine hours were associated with a higher SV (beta 5.86, 95%-CI [2.57; 9.16], p < 0.001, beta 0.19, 95%-CI [− 0.28; 0.75], p = 0.881, and beta 0.01, 95%-CI [− 0.05; 0.06], p = 0.887, respectively). In contrast, a higher temperature, higher humidity, and a higher number of foreign-born residents per 100,000 inhabitants resulted in a lower SV (beta − 0.60, 95%-CI [− 1.06; − 0.13], p = 0.012, beta − 0.25, 95%-CI [− 0.67; 0.18], p = 0.258, and beta − 0.01, 95%-CI [− 0.006; − 0.004], p < 0.001, respectively). Adjusted *R*^*2*^ of model summary was 0.316.

## Discussion

Our analysis of sarcoidosis-related web search data showed that in Swedish counties, the highest number of search queries was observed in the spring and autumn. Västerbotten, Jämtland, and Västernorrland the counties with the highest number of new sarcoidosis cases per 100,000 inhabitants, had more searches per 100,000 inhabitants compared to the other counties. A positive correlation was found between web search queries and monthly incidence on country level showing that web search queries can serve as a proxy for the incidence of sarcoidosis. Further, we found that counties with a higher proportion of male inhabitants, lower average temperature, and fewer foreign-born residents have significantly more sarcoidosis-related searches.

Previous studies using web search data have also demonstrated positive correlations with various disease registry data (e.g., for conjunctivitis^24^, coronary heart disease^19^, and cancer incidence and mortality^20,21^). However, the correlations observed in our study were weaker compared to those of previous studies using web search data. This may be because sarcoidosis is a rare disease with not as readily available high-quality information on the internet^25^ and low exposure in the media compared to the previously studied diseases^26^. Additionally, it should be noted that the date of the ICD-coded visit listing sarcoidosis may be different than when the patient begins searching for information on the internet if they are suspected of having sarcoidosis, but it is not confirmed until the visit. This aspect should be taken into account in further investigations. Nevertheless, web search data seem to play an important role in gathering information about rare diseases for affected individuals^15^. Analysing web search data for sarcoidosis therefore remains a feasible and inexpensive solution to disease surveillance.

Our study showed a relationship between the geographical variation in the number of searches. Geographical differences in the prevalence of sarcoidosis between different areas of one country is a phenomenon observed worldwide^27–29^. For Sweden, the highest sarcoidosis prevalence was reported for the less densely populated north-western regions, and it was discussed that these differences may be caused by the population genetics of the Swedish counties, with higher ethnic diversity in the southern urbanized counties^6^. This theory is in agreement with our study, as a higher population density and number of foreign-born residents were associated with fewer searches for sarcoidosis. However, foreign-born residents can also search online in another language, which should be taken into account when interpreting the results.

The higher prevalence of sarcoidosis observed in northern counties could be due to decreased exposure to sunlight and thereby a vitamin D deficiency may be associated with sarcoidosis^8^. Moreover, exposure to humidity and lower temperature also appear to be associated with sarcoidosis, which could explain the observed geographical variations^30^. Such an association was only observed to a small degree in our study, with a lower temperature being associated with more search queries. Thus, as only 30% of the variation in search behaviour could be explained by the assessed variables including age and sex, there must be additional variables that influence sarcoidosis-related search behaviour.

A recent study indicated differences in sarcoidosis diagnoses and treatment among different Swedish healthcare regions, which may suggest differences in awareness of the disease^31^. Differences in diagnosis and treatment do not appear to match the differences in prevalence in Sweden^31^. However, it was observed that a better supply of general practitioners and outpatient specialists was associated with a higher number of searches in Germany^17^. Regional differences in Sweden’s healthcare system and utilization^32^ may therefore explain further variations in the search behaviour.

We identified a seasonal pattern that showed a higher number of sarcoidosis-related search queries during the spring. This is in accordance with prior studies assessing sarcoidosis-related web search data^22,23^ and various epidemiological studies that demonstrated sarcoidosis occurring more commonly during the spring^8,10^. One explanation for this seasonality is that sarcoidosis may be triggered by organic bioaerosols, predominately pollen, that are more common during springtime^8^. It is also possible that there is a connection between public holidays in Sweden (July/August and December/January) and the associated shifts in diagnosis. Signs and symptoms tend to be overlooked and left unaddressed until after the vacation period^33,34^. Other epidemiological studies also reported that the prevalence of sarcoidosis is associated with certain industrial organic and inorganic dust^8,35^; agents that are found in abundance in the work environment of miners, firefighters, and agricultural workers^9,35–37^, occupational groups which show a higher risk for sarcoidosis, a factor that should be considered in future analyses.

Despite the absence of identified COVID-19-related keywords, the elevated SV observed in 2020 can be attributed in part to the pandemic’s influence. This phenomenon may stem from factors such as decreased access to medical appointments for symptom clarification, medical inquiries, and support provision for affected individuals^38^.

The reliability of the internet search data for sarcoidosis in Sweden may be limited, as it only identified 30 keywords with an overall SV of 123,556 searches per 100,000 inhabitants, while for Germany 433 keywords associated with sarcoidosis with a SV of 3,068,200 per 100,000 inhabitants^22^ were identified despite a similar market share of Google (> 90%^39^). Moreover, the accuracy of Swedish websites compared to English or other language websites may differ, potentially affecting the study's results. In addition, internet search data and disease incidence did not match in time due to limited data availability. Analyses of Google search queries are limited to individuals who have internet access and use this search engine. Although 98% of Sweden’s households had access to the Internet and 94% of the population used it in 2018^40^ (93% of Internet users using Google as a search engine^39^), only including data collected by Google excludes information from non-users. Although the elderly are increasingly using the internet, younger people still use the internet more frequently in Sweden^40^. We only included data from searches with the Swedish language preferred and are therefore missing searches done in other languages. No statements about individual users can be made since Google Ads do not provide information about user demographics. However, we observed correlations between the percentages of male inhabitants and the number of foreign-born residents, which indicate the influence of demographics on search behaviour. Also, educational level and communication habits among affected individuals can impact online search behaviour and should be considered in future research. It is essential to recognize, however, that our study adopts an ecological design, and caution must be exercised to avoid the ecological fallacy when interpreting these findings^41^. In addition, it must be considered that the searches do not necessarily only originate from affected individuals but also from their relatives, physicians, medical and science students, or national visitors to medical congresses interested in sarcoidosis. We did not have information on searches for Löfgren syndrome, which would be interesting to include in future studies to examine whether trends and relationships are different by sarcoidosis phenotype. Google provides automatic completion of search suggestions, which could also bias a person’s search behaviour. Moreover, the data provided by Google Ads are based only on monthly estimations instead of exact numbers. Thus, the estimations may be overestimated for the individual counties and underestimated for Sweden as a whole^42^.

In summary, our study demonstrated a correlation between Google searches for sarcoidosis and sarcoidosis incidence across Sweden. We characterized seasonal trends and identified geographical variations that may serve as a point of comparison or a standard for future studies or analyses. Moreover, we found that a higher number of searches was correlated with lower temperature and a lower proportion of foreign-born residents.

## Conclusion

Analysis of Internet search data is a novel approach to detecting unmet medical needs, gauging public interest in sarcoidosis, and identifying high-risk groups and new risk factors. However, further analyses are needed with internet search and incidence data referring to the same period, and future studies should focus on more environmental and health-specific aspects to evaluate the specificity and sensitivity of the web search data.

### Supplementary Information

Supplementary Tables.

## Data Availability

The datasets used during the current study are available from the corresponding author on reasonable request.
