# Supplementary material for: Identifying sarcoidosis trends using web search and real-world data in Sweden: a retrospective longitudinal study
Source: Sci Rep. 2024 Aug 20;14:19260. doi: 10.1038/s41598-024-69223-8 (PMC11335935; doi:10.1038/s41598-024-69223-8)
Supplement: Supplementary file 1 — Supplementary Tables. [file 41598_2024_69223_MOESM1_ESM.pdf]

Identifying Sarcoidosis Trends using Web Search and Real-World Data in Sweden from 2007-2020

Table S1 Inhabitants in Sweden by county and year obtained from Statistics Sweden (Statistiska Centralbyrån, scb.se).

	2017	2018	2019	2020
**01 Stockholm County**	2,308,143	2,344,124	2,377,081	2,391,990
**03 Uppsala County**	368,971	376,354	383,713	388,394
**04 Södermanland County**	291,341	294,695	297,540	299,401
**05 Östergötland County**	457,496	461,583	465,495	467,158
**06 Jönköping County**	357,237	360,825	363,599	365,010
**07 Kronoberg County**	197,519	199,886	201,469	202,263
**08 Kalmar County**	243,536	244,670	245,446	246,010
**09 Gotland County**	58,595	59,249	59,686	60,124
**10 Blekinge County**	159,371	159,684	159,606	159,056
**12 Skåne County**	1,344,689	1,362,164	1,377,827	1,389,336
**13 Halland County**	324,825	329,352	333,848	336,748
**14 Västra Götaland County**	1,690,782	1,709,814	1,725,881	1,734,443
**17 Värmland County**	280,399	281,482	282,414	282,885
**18 Örebro County**	298,907	302,252	304,805	305,643
**19 Västmanland County**	271,095	273,929	275,845	277,141
**20 Dalarna County**	286,165	287,191	287,966	287,676
**21 Gävleborg County**	285,637	286,547	287,382	287,502
**22 Västernorrland County**	245,968	245,453	245,347	244,554
**23 Jämtland County**	129,806	130,280	130,810	131,155
**24 Västerbotten County**	268,465	270,154	271,736	273,192
**25 Norrbotten County**	251,295	250,497	250,093	249,614

Table S2 Keywords related to the Swedish search term for sarcoidosis in Swedish and their English translation as well as their category according to the qualitative analysis (n=30)

Swedish keyword	English translation	Category
sarkoidos	sarcoidosis	manifestations and forms
lungsarkoidos	lung sarcoidosis	manifestations and forms
neurosarkoidos	neurosarcoidosis	manifestations and forms
sarkoidos hud	sarcoidosis skin	manifestations and forms
sarkoidos lungor	sarcoidosis lungs	manifestations and forms
obehandlad sarkoidos	untreated sarcoidosis	treatment
hjärtsarkoidos	cardiac sarcoidosis	manifestations and forms
sarkoidos symtom	sarcoidosis symptom	symptoms
sarkoidos internetmedicin	sarcoidosis internet medicine*	information
sarkoidos ögon	sarcoidosis eyes	manifestations and forms
akut sarkoidos	acute sarcoidosis	manifestations and forms
lungsjukdomar sarkoidos	lung disease sarcoidosis	manifestations and forms
hiluslymfom	hilar lymphadenopathy	manifestations and forms
sarkoidos hjärta	sarcoidosis heart	manifestations and forms
sarkoidos cellgifter	sarcoidosis cytotoxic drugs	treatment
sarkoidos behandling	sarcoidosis treatment	treatment
sarkoidos i hjärnan symtom	sarcoidosis of the brain symptom	manifestations and forms
vad är sarkoidos	what is sarcoidosis	information
sarkoidosen	sarcoidoses	manifestations and forms
lungsjukdom sarkoidos	lung disease sarcoidosis	manifestations and forms
vad är neurosarkoidos	what is neurosarcoidosis	information
kronisk sarkoidos	chronic sarcoidosis	manifestations and forms
sarkoidos hjärta symptom	sarcoidosis heart symptom	symptoms
sarkoidos lungröntgen	sarcoid lung X-ray	visualisation
kutan sarkoidos	cutaneous sarcoidosis	manifestations and forms
sarkoidos i hjärtat	sarcoidosis of the heart	manifestations and forms
sarkoidos lymfkörtlar	sarcoidosis lymph nodes	manifestations and forms
sarkoidos leder	sarcoidosis joints	treatment
neurosarkoidos symtom	neurosarcoidosis symptom	manifestations and forms
pulmonell sarkoidos	pulmonary sarcoidosis	manifestations and forms

* Internet medicine is a knowledge database for doctors with concentrated overviews for diagnosis and treatment of medical conditions.

Table S3 Correlation between variables entered in the multiple logistic regression model.

		Mean age	Male inhabitants	Population density	Foreign-born persons	Sarcoidosis diagnoses	Humidity (%)	Temperature (°C)	Sunshine duration (h)
Mean age	r*	1	*.093*	*-.170*	*-.112*	.020	.029	-.013	.000
	p	.	*.003*	*<.001*	*<.001*	.530	.353	.685	.998
Male inhabitants	r		1	*-.120*	*-.152*	*.098*	.052	-.048	-.033
	p		.	*<.001*	*<.001*	*.002*	.099	.129	.229
Population density	r			1	***.696***	*-.076*	-.035	*.013*	.033
	p			.	***<.001***	*.015*	.262	*.001*	.298
Foreign-born persons	r				1	*-.069*	-.067	*-.069*	.005
	p				.	*.029*	0.520	*.029*	.890
Sarcoidosis diagnoses	r					1	*-.020*	*-.094*	-.002
	p					.	.535	*.033*	.951
Humidity (%)	r						1	*.284*	*.290*
	p						.	*<.001*	*<.001*
Temperature (°C)	r							1	*.299*
	p							.	*<.001*
Sunshine (h)	r								1
	p								.

*Spearman correlation coefficient
